# Oral Health Knowledge of Periodontal Disease among University Students

**DOI:** 10.1155/2013/647397

**Published:** 2013-03-20

**Authors:** Bader K. Al-Zarea

**Affiliations:** Faculty of Dentistry, Al-Jouf University, P.O. Box 2232, Aljouf, Sakaka 42421, Saudi Arabia

## Abstract

*Objectives*. The aim of this study was to evaluate levels of oral health knowledge of periodontal disease among nondental university students. *Materials and Methods*. Two hundred and fifty university students (mean age 20.1 years ± 2.5) were recruited into this study. The participants completed a structured questionnaire during a personal interview. The questionnaire consisted of items to assess participants' personal data (age, gender, level of study, and specialty) and oral heath knowledge related to periodontal disease. Statistical significance was based on probability values of less than 0.05. *Results*. Participants showed poor knowledge of causes, signs, symptoms, and preventive measures of gum disease. The level of study had no relationship with students' knowledge of the initiating factors of periodontal disease (*P* < 0.05), but had a significant relationship with the knowledge of periodontal disease's signs, preventive measures, and relations to general health and systemic disease (*P* < 0.05). Students from scientific disciplines had more knowledge of periodontal disease's causes, preventive measures, and relations to general health and systemic disease (*P* < 0.05) than those from humanity disciplines. *Conclusions*. There were significant differences in oral health knowledge regarding periodontal disease between students from different levels of studies and different disciplines.

## 1. Introduction

Periodontal disease, including gingivitis and periodontitis, is considered to be one of the most common diseases among population and, if left untreated, can lead to tooth loss [1]. The main cause of periodontal disease is bacterial plaque although many other factors such as hormonal changes, diabetes, poor nutrition, smoking, and stress may affect the initiation and progression of gingival and periodontal diseases [2]. The development of the common periodontal diseases depends mainly on human behavior, and the control of these diseases is greatly supported by the fact that the etiological factors are well documented [3].

Effective plaque control is an essential part in the treatment of inflammatory periodontal diseases [4]. Many studies showed that effective plaque control for each person cannot be achieved without interactive motivation that includes educational and informative knowledge for the patient about periodontal diseases, their initiating factors, and the major role of dental plaque as the initiating cause for inflammatory periodontal changes [4, 5]. Nettleton [6] emphasized the need for offering patients accurate information so that they can make an educated decision about their own behavior and actions.

In Arab world, although studies were carried-out regarding the prevalence of gingival and periodontal diseases [7], as well as periodontal disease knowledge and awareness among adults and children [8], none were carried out among university students to assess their knowledge towards gingival and periodontal health, except for three studies which assessed the dental and oral health attitudes and behaviors among dental-field-related university students [9–11].

Quteish Taani [12] showed that 25% of adults suffered bleeding gums on brushing and around the same percentage suffered bad breath. Nearly 40% of adults believed that they had periodontal disease. However, the knowledge of periodontal problems was found to be poor among adults. These data indicate that development and implementation of well-structured dental health education programs are needed to improve and maintain suitable oral health standards among the population.

In Saudi, the oral health system is in a transitional developmental stage, and systemic data collection is needed to plan oral health care for the public. Comprehensive preventive programs for oral health care are still lacking in Saudi, and more dental health education is needed to improve oral health standards among Saudi population.

By reviewing the available literature, it appeared that we lack data concerning the effect of the student's level of studies and study discipline (i.e., humanities or scientific faculties) on oral health knowledge. These factors might have a great influence on student's knowledge through the types of courses they submit, and the effects of these courses on the information which could be delivered to them, or from their colleagues in different faculties at the university.

Little is known about the oral health knowledge among university students from developing countries such as Saudi in comparison with those from developed countries although such knowledge is an indication of the efficacy of applied dental health education programs. 

Oral health knowledge related to periodontal diseases has a major role in the treatment and prevention of the disease among children, adolescents, and adults including university students [13, 14]. Consequently, the purpose of this study was to investigate the knowledge about periodontal oral health and the knowledge of the causes of inflammatory periodontal diseases among university students. This study provides data for future research and allows comparisons with university students' oral health knowledge in other nations.

## 2. Materials and Methods 

Two hundred and fifty nondental students were recruited into this cross-sectional study after being randomly selected from the university students who attended the university campus during the second semester of the academic year 2011/2012. The recruited students were all from the levels of first and final year. The study was approved by University of Al-Jouf, and participants consent was obtained before being recruited into the study. 

A previously prepared structured questionnaire was distributed to the participants through personal interview by the researcher. All participants were provided with full explanation of the study and the questionnaire. Medical and dental terms of the questionnaire related to causes, signs, and symptoms of periodontal diseases were also explained to them during the study. Once completed, each questionnaire was double-checked to make sure that all the items were answered and participants were requested to complete any missing data. The data were processed by the computer after auditing, reviewing, and coding the completed questionnaires for data processing and analysis.

### 2.1. Questionnaire Design

A preliminary questionnaire with close ended questions was developed. The questionnaire included items regarding causes, signs, symptoms, and preventive measures of periodontal disease. Each question was given one correct statement and the other statements were wrong, and the participant responded to the statement by selecting one of three responses, namely, yes, no, or I do not know. 

To test its validity the questionnaire was presented to three arbitrators from the teaching staff of the Faculty of Dentistry, The University of Al-Jouf; accordingly, reformulation of some of the terminology was carried out and some of the answers that are common errors were added.

The final form of the questionnaire (Table 1) included data relevant to the characteristics of the participants, that is, gender, year of study, and study discipline. It also consisted of seven items to assess the students' knowledge regarding the periodontal disease causes, signs, symptoms, preventive measures, and relations to general heath.

To test its reliability, the questionnaire was distributed to ten students in the Faculty of Dentistry. The students completed the test twice on two occasions separated by 5 days, and the reproducibility of the answers ranged between 90 and 100% which indicated adequate reliability and stability of the questionnaire. It took most of the participants 5–7 minutes to complete the questionnaire. 

### 2.2. Statistical Analysis

The data was processed and analyzed by means of computerized SAS statistical package. Frequency tables, percentages, and cross-tables were generated. Chi-squared test was used to identify significant relations and differences between oral health knowledge and gender, level of study, and discipline of study. Statistical significance was based on probability values of less than 0.05 (*P* < 0.05). 

## 3. Results

The final study sample consisted of 250 male nondental university students (mean age 21 years ± 2).  Table 2 presents the distribution of the study sample according level of study and the study discipline (humanities or scientific studies). More than half of the sample were first year students (54.8%) and 40% of the participants were studying in scientific faculties of the University of Al-Jouf. 

Table 3 summarizes the distribution of participants' knowledge of causes, signs, and preventive measures of periodontal disease according the level of the study. Similar levels of knowledge regarding the cause of periodontal disease (bacterial plaque) were reported by students from both study levels (*P* = 0.9). However, final year students were more knowledgeable than first year students regarding gingival bleeding being the most important sign of periodontal disease (*P* = 0.0004). Also, final year students had better knowledge of the preventive measures for periodontal disease than first year students (*P* = 0.0012).

Table 4 demonstrates the distribution of the participants' knowledge of causes, signs, and preventive measures of periodontal disease according the discipline of the study. Students from scientific faculties had better knowledge of the causes and preventive measures of periodontal disease than those from humanities faculties (*P* = 0.0001). Although students from scientific faculties had slightly better knowledge of the signs of periodontal disease, the difference was not significant (*P* = 0.1).

Final year students had better knowledge of the relationship between periodontal disease, and general health than first year students (*P* < 0.05) (Table 5).

Students from higher study levels had better knowledge of the relationship between periodontal disease on one hand and each of smoking, heart disease, and diabetes on the other hand (*P* = 0.0001). Also, final year students had better knowledge of the effect of mouth wash in reducing bad breath than first year students (*P* = 0.0001) (Table 5).

Students of scientific faculties had better knowledge of the relationship between smoking and diabetes on one hand and periodontal disease on the other hand (*P* = 0.02 and 0.0001 resp.) than students of humanities faculties (Table 6). Also, students of scientific faculties had better knowledge of the effect of mouth wash in reducing bad breath as well as better knowledge of the relationship between heart disease and periodontal disease (*P* = 0.0001) than students of humanities faculties.

## 4. Discussion

The aim of this study was to compare the oral health knowledge of nondental students of different disciplines and study levels. The literature lacks studies about oral health knowledge regarding periodontal disease among nondental Saudi university students. This study is of prime importance in this field as it is the first one to explore this area among nondental students and among Saudi university students. University students are a good representative sample for the population since they reflect education, socioeconomic conditions, acculturation, psychological stress, and culture, which can affect their oral health behavior and status.

Higher levels of studies had better knowledge of periodontal disease signs, preventive measures, and relations to general health, and this might be due to receiving more dental health care and thus know more about periodontal disease. periodontal disease. Other studies proved that within the same specialty the dental health knowledge and attitudes became more positive with the increase of level of education [15, 16].

The finding that students from scientific faculties had better knowledge of periodontal disease causes, signs, preventive measures, and relations to general health than students from humanity disciplines might be explained by those students from scientific disciplines might be more interested with health related-issues and thus get some kind of general health education that could involve some oral health issues and their relations to general health. So, the variations in favorability of oral health knowledge in the study sample reflect the students' interests, education, and their curriculum.

It does worth comparing the oral health knowledge between different specialties from different countries, and this might cast the light on the weaknesses in the oral health education programs and allow improving such programs. When the results of this study were compared to European populations [17–20], European adults demonstrated better dental knowledge than their Saudi peers.

We believe that oral care educational needs to improve oral health knowledge do exist in many developing countries especially in the third world. The results of this study might help to evaluate the efficacy of public education programs in future. The results also indicate that potential parents' (university students) education must be included in any national program that promotes preventive oral care in schools as well as in other oral health educational program aimed at the general public [21].

Due to political and economical changes in Baltic and Eastern European countries, dental health care has been given greater weight, and a decrease in caries prevalence was recorded [18, 22]. Comprehensive oral health educational programs were directed toward the professionals and the public and aimed at adults and young people. Meanwhile, the governmental legislations and financial support assisted the implication of such programs and thus maximized the gains. Political as well as economic reforms led to the participation of the UN and international organizations in the process of reforming the health sector and thus boosted the standards of oral health care. The experience of Eastern European countries might be relevant to Saudi and implemented by the dental health authorities here because Saudi is witnessing very promising economic and social reforms.

Lack of both parents and children oral health education might also explain the findings of this study [12, 23]. Poor oral health knowledge among the participants in this study coincided with findings from the previous studies that reported lack of acceptable levels of knowledge and awareness of periodontal problems among Saudi adults [12, 23].

During the last decade extensive efforts have been made by the dental schools in Saudi in an attempt to improve the periodontal knowledge and practice of the dental personnel, but still these efforts are not enough to raise the standards of professional periodontal practice among dentists which directly affects the public [12, 23]. Consequently, dental health education programs that aim to improve oral health knowledge and practice among the public are very important. Improving public awareness of periodontal health should be an essential public health goal.

In many developing countries, there is no emphasis on dental health care during primary, secondary, and high school teaching. Knowledge and awareness concerning some oral health subjects (such as periodontal disease) are still poor, and more dental health education is needed to improve oral health [12]. Quteish Taani [12] showed that high percentage of adults reported gum bleeding on brushing, bad breath, and being irregular attendees to the dentist. In another study, he showed that around 80% of the subjects attended the dentist only in an emergency [24]. Furthermore, nondental university students receive no oral health education at all during their university study, and their curriculums contain no information regarding oral health education.

The abovementioned findings in the literature might explain why many participants demonstrated poor knowledge of periodontal disease signs, causes, preventive measures, and relations to general health, systemic disease, and smoking. 

These findings make it necessary to carry out more research on Saudi oral health knowledge and behavior as well as to improve their oral health care education systems. Further studies are required to assess oral health knowledge and behavior among Saudi female university students. This study was only carried out on males since the tested disciplines does not include females. This is one of the limitations in this study.

Emphasis on dental health care and education should be developed and maintained during early school education in order to improve the oral health knowledge of adults later on. Oral health education should also be included and emphasized in university curriculums for nondental students during their university study. It is well known that the oral health of parents reflects on their children and that their attitudes and knowledge affect their children. Hence, educating adults and university students seems among the means to improve the oral health knowledge and behavior of the nation in future. Therefore, preuniversity and university curriculum and education about dental health care might be an important factor that can influence the oral health knowledge and attitudes of students not related to dental field.

## 5. Conclusions

This study demonstrated that there were significant differences in oral health knowledge among students from different levels of studies and different disciplines. The findings might reflect the difference in students' experience, attitudes, behavior, and education. 

Students from scientific faculties had better knowledge of periodontal disease causes, signs, preventive measures, and relations to general health than students from humanity disciplines. Students from higher levels of studies had better knowledge of periodontal disease signs, preventive measures, and relations to general health than those from lower levels of study. 

Finally, oral health education should be included and emphasized in university curriculums for nondental students during their university study.

## Figures and Tables

**Table 1:** Constructed questionnaire items and responses used in this study.

Questions	Yes	No	I do not know
(1) What is the initiating factor of periodontal disease?
Bacterial plaque
Dental calculus
Malnourishment
Hereditary
Diabetes
Infection
(2) What is the most indicating sign of periodontal disease?
Gingival bleeding
Gingival swelling
Gingival redness
Bad breath
(3) What is the effective measure to prevent periodontal disease?
Use toothbrush and dental floss
Good nourishment
Regular visits to the dentist
(4) Do we get rid of bad breath by using mouthwashes?
(5) Is there any relation between gum disease and diabetes?
(6) Is there any relation between gum disease and heart diseases?
(7) Is there any relation between gum disease and smoking?

**Table 2:** Frequency distribution of the sample according to faculty and year of study (*N* = 250).

Variable	*n*	(%)
Year of study		
First year	137	(54.8)
Final year	113	(45.2)
Faculty		
Humanities	150	(60)
Scientific	100	(40)

**Table 3:** Distribution of responses to periodontal health knowledge questions by year of study.

Questions	First year (*n* = 137)			Final year (*n* = 113)			*P* value (*χ* ^2^ test)
	Yes (%)	No (%)	Do not know (%)	Yes (%)	No (%)	Do not know (%)
(1) What is the initiating factor of periodontal disease?							
Bacterial plaque*	62	15	23	66	16	18	0.9083
Dental calculus	50	20	30	55	30	15	
Malnourishment	60	18	22	57	23	20	
Hereditary	57	20	23	60	30	10	
Diabetes	19	43	38	65	20	15	
Infection	20	70	10	17	50	33	
(2) What is the most indicating sign of periodontal disease?							
Gingival bleeding*	70	10	20	94	4	2	0.0004
Gingival swelling	65	25	10	80	12	8	
Gingival redness	60	27	13	75	11	14	
Bad breath	40	23	37	61	37	2	
(3) What is the effective measure to prevent periodontal disease?							
Use toothbrush and dental floss*	72	27	1	90	6	4	0.0012
Good nourishment	87	8	5	70	16	14	
Regular visits to the dentist	81	17	2	82	10	8	

*The correct answer.

**Table 4:** Distribution of responses to periodontal health knowledge questions by faculty.

Questions	Humanities faculties (*n* = 150)			Scientific faculties (*n* = 100)			*P* value (*χ* ^2^ test)
	Yes (%)	No (%)	Do not know (%)	Yes (%)	No (%)	Do not know (%)
(1) What is the initiating factor of periodontal disease?							
Bacterial plaque*	50	16	34	95	2	3	0.0001
Dental calculus	64	20	16	89	9	2	
Malnourishment	56	15	29	86	9	5	
Hereditary	24	40	36	88	5	7	
Diabetes	10	46	44	93	3	4	
Infection	15	55	35	15	83	2	
(2) What is the most indicating sign of periodontal disease?							
Gingival bleeding*	80	10	10	90	5	5	0.1
Gingival swelling	68	20	12	92	6	2	
Gingival redness	68	14	18	89	10	1	
Bad breath	25	40	35	93	3	4	
(3) What is the effective measure to prevent periodontal disease?							
Use toothbrush and dental floss*	75	10	15	96	4	0	0.0001
Good nourishment	80	10	10	87	4	9	
Regular visits to the dentist	80	8	16	92	1	7	

*The correct answer.

**Table 5:** Distribution of responses to oral and general health knowledge by year of study (*n* = 250: first year 137 and final year 113).

Questions	First year (%)	Final year (%)	*P* value (*χ* ^2^ test)
(1) Do we get rid of bad breath by using mouthwashes?			
(a) Yes*	45	90	
(b) No	30	6	0.0001
(c) I do not know	25	4	
(2) Is there any relation between gum disease and diabetes?			
(a) Yes*	52	83	
(b) No	20	7	0.0001
(c) I do not know	28	10	
(3) Is there any relation between gum disease and heart diseases?			
(a) Yes*	2	22	
(b) No	82	32	0.0001
(c) I do not know	16	46	
(4) Is there any relation between gum disease and smoking?			
(a) Yes*	78	94	
(b) No	8	2	0.0001
(c) I do not know	14	4	

*The correct answer.

**Table 6:** Distribution of responses to oral and general health knowledge by faculty (*n* = 250: humanities 150 and scientific 100).

Questions	Humanities (%)	Scientific (%)	*P* value (*χ* ^2^ test)
(1) Do we get rid of bad breath by using mouthwashes?			
(a) Yes*	58	80	
(b) No	20	4	0.0001
(c) I do not know	22	16	
(2) Is there any relation between gum disease and diabetes?			
(a) Yes*	50	84	
(b) No	20	2	0.0001
(c) I do not know	30	14	
(3) Is there any relation between gum disease and heart diseases?			
(a) Yes*	10	15	
(b) No	50	60	0.0001
(c) I do not know	40	25	
(4) Is there any relation between gum disease and smoking?			
(a) Yes*	60	85	
(b) No	8	5	0.001
(c) I do not know	32	10	

*The correct answer.
